# Supplementary material for: Laser Acupuncture versus Liraglutide in Treatment of Obesity: A Multi-Institutional Retrospective Cohort Study
Source: Healthcare (Basel). 2024 Jun 26;12(13):1279. doi: 10.3390/healthcare12131279 (PMC11241425; doi:10.3390/healthcare12131279)
Supplement: Supplementary file 1 [file healthcare-12-01279-s001.zip › healthcare-2975893-supplementary.pdf]

Supplementary Table S1. Summary of acupoints used for laser acupuncture in the present study.
* Bahr refers to a specific frequency type used in laser acupuncture settings.

Name	WHO standard name	Location	Mode
Stomach point		Located at the end of the crus of the helix in the cavum concha. It is the area transformed by the crus of the helix to the border of the lower antihelix crus.	Bahr* 6, 7
Hunger point		Located on the lower part of the tragus in the direction of the transitional fold near the facial skin.	Bahr 6, 7
Tian-shu	ST25	Located on the upper abdomen, 2 B-cun lateral to the center of the umbilicus.	Bahr 2
Shui-dao	ST28	Located on the lower abdomen, 3 B-cun inferior to the center of the umbilicus, 2 B-cun lateral to the anterior median line.	Bahr 2
Feng-long	ST40	Located on the anterolateral aspect of the leg, the lateral border of the tibialis anterior muscle, 8 B-cun superior to the prominence of the lateral malleolus.	Bahr 2
Da-heng	SP15	Located on the upper abdomen, 4 B-cun lateral to the umbilicus	Bahr 2
Shui-fen	CV9	Located on the upper abdomen, 1 B-cun superior to the umbilicus, on the anterior median line.	Bahr 3

Supplementary Table S2. Diagnostic codes used in the study.

Diseases	ICD-9-CM codes	ICD-10 codes
Hypertension	4010, 4011, 4019	I10
Dyslipidemia	2722, 2724, 2729	E782, E784, E785
Ischemic heart diseases	4111, 4130, 4131, 4139, 410, 4295, 4296, 42971, 42979, 4110, 41181, 41189, 412, 414	I20, I201, I208, I209, I21, I211, I212, I213, I214, I219, I22, I221, I228, I229, I23, I231, I232, I233, I234, I235, I236, I238, I24, I241, I248, I249, I25, I251, I252, I255, I256, I258, I259
CVD	36234, 430, 431, 432, 433, 434, 435, 436, 437, 438	G45, G46, H340, I60, I61, I62, I63, I64, I65, I66, I67, I68, I69
MI	410, 412	I21, I22, 252
CHF	39891, 40201, 40211, 40291, 40401, 40403, 40411, 40413, 40491, 40493, 4254, 4255, 4256, 4257, 4258, 4259, 428	I099, I110, I130, I132, I255, I420, I425, I426, I427, I428, I429, I43, I50, P290
PVD	0930, 4373, 440, 441, 4431, 4432, 4433, 4434, 4435, 4436, 4437, 4438, 4439, 5571, 5579	0930, 4373, 440, 441, 4431, 4432, 4433, 4434, 4435, 4436, 4437, 4438, 4439, 5571, 5579
Dementia	290, 2941, 3312	F00, F01, F02, F03, F051, G30, G311
Rheumatism	4465, 7100, 7101, 7102, 7103, 7104, 7140, 7141, 7142, 7148, 725	M05, M06, M315, M32, M33, M34, M351, M353, M360
Ulcer	531, 532, 533, 534	K25, K26, K27, K28
Hemiplegia	3341, 342, 343, 3440, 3441, 3442, 3443, 3444, 3445, 3446, 3449	G041, G114, G801, G802, G81, G82, G830, G831, G832, G833, G834, G839
Renal disease	40301, 40311, 40391, 40402, 40403, 40412, 40413, 40492, 40493, 582, 5830, 5831, 5832, 5833, 5834, 5835, 5836, 5837, 585, 586, 5880	V420, V451, V56, I120, I131, N032, N033, N034, N035, N036, N037, N052, N053, N054, N055, N056, N057, N18, N19, N250, Z490, Z491, Z492, Z940, Z992
MSLD	4560, 4561, 4562, 5722, 5723, 5724, 5725, 5726, 5727, 5728	I850, I859, I864, I982, K704, K711, K721, K729, K765, K766, K767
CPD	4168, 4169, 490, 491, 492, 493, 494, 495, 496, 500, 501, 502, 503, 504, 05, 5064, 5081, 5088	I278, I279, J40, J41, J42, J43, J44, J45, J46, J47, J60, J61, J62, J63, J64, J65, J66, J67, J684, J701, J703
MLD	07022, 07023, 07032, 07033, 07044, 07054, 0706, 0709, 570, 571, 5733, 5734, 5738, 5739	V427, B18, K700, K701, K702, K703, K709, K713, K714, K715, K717, K73, K74, K760, K762, K763, K764, K768, K769, Z944

DM with chronic disease	2504, 2505, 2506, 2507	E102, E103, E104, E105, E107, E112, E113, E114, E115, E117, E122, E123, E124, E125, E127, E132, E133, E134, E135, E137, E142, E143, E144, E145, E147
DM without chronic disease	2500, 2501, 2502, 2503, 2508, 2509	E100, E101, E106, E108, E109, E110, E111, E116, E118, E119, E120, E121, E126, E128, E129, E130, E131, E136, E138, E139, E140, E141, E146, E148, E149
Malignancy	140, 141, 142, 143, 144, 145, 146, 147, 148, 149, 150, 151, 152, 153, 154, 155, 156, 157, 158, 159, 160, 161, 162, 163, 164, 165 , 166, 167, 168, 169, 170, 171, 172, 174, 175, 176, 177, 178, 179, 180, 181, 182, 183, 184, 185, 186, 187, 188, 189, 190, 191, 192, 193, 194, 1950, 1951, 1952, 1953, 1954, 1955, 1956, 1957, 1958, 200, 201, 202, 203, 204, 205, 206, 207, 208, 2386	C00, C01, C02, C03, C04, C05, C06, C07, C08, C09, C10, C11, C12, C13, C14, C15, C16, C17, C18, C19, C20, C21, C22, C23, C24, C25, C26, C30, C31, C32, C33, C34, C37, C38, C39, C40, C41, C43, C45, C46, C47, C48, C49, C50, C51, C52, C53, C54, C55, C56, C57, C58, C60, C61, C62, C63, C64, C65, C66, C67, C68, C69, C70, C71, C72, C73, C74, C75, C76, C81, C82, C83, C84, C85, C88, C90, C91, C92, C93, C94, C95, C96, C97
Cancer	196, 197, 198, 199	C77, C78, C79, C80
AIDS	042, 043, 044	B20, B21, B22, B24
Chronic hepatitis	57149, 57140	K738, K739
Fatty liver	5718	K760
Non-alcoholic steatohepatitis	5718, 5733	K7581
Hemorrhagic stroke	430, 431, 432	I60, I601, I602, I603, I604, I605, I606, I607, I608, I609, I61, I611, I612, I613, I614, I615, I616, I618, I619, I62, I621, I629
Ischemic stroke	433, 434	I63, I631, I632, I633, I634, I635, I636, I638, I639, I64, I65, I651, I652, I653, I658, I659, I66, I661, I662, I663, I664, I668, I669
Hypertensive encephalopathy	4372	I674

Abbreviations: AIDS, acquired immunodeficiency syndrome; CPD, chronic pulmonary disease; CVD, cerebrovascular disease; DM, diabetes mellitus; ICD-9-CM, International Classification of Diseases, 9th Revision, Clinical Modification; ICD-10, International Classification of Diseases, 10th Revision; MI, myocardial infarction; MLD, moderate liver disease; MSLD moderate to severe liver disease; PVD, peripheral vascular disease

Supplementary Table S3. Changes in primary endpoints and the risk of adverse events between baseline and day 180 (balanced with IPTW).

	LA users	Liraglutide users	p-value
**Changes in body weight**			
Body weight (kg)	-5.92±4.4	-2.34±2.97	<0.001
% of body weight	6.84%	2.65%	<0.001
Loss >5% of body weight (%)	60.7%	22.8%	<0.001
Loss >10% of body weight (%)	24.0%	4.3%	<0.001
Loss >15% of body weight (%)	4.8%	1.1%	0.043
**Changes in body mass index**	-2.21±1.63	-0.94±1.19	<0.001
**Incidence of adverse events**			
Hypertension	0.00%	2.86%	0.025
Ischemic heart disease	0.00%	0.64%	0.290
Hemorrhagic stroke	0	0	-
Ischemic stroke	0	0	-

Abbreviations: IPTW, inverse probability of treatment weighting; LA, laser acupuncture

Supplementary Table S4. Changes in primary endpoints and the risk of adverse events between baseline and day 180 (balanced with 1:1 PSM).

	**LA users (n=37)**	**Liraglutide users (n=37)**	**p-value (between group)**
**Changes in body weight**			
Body weight (kg)	−5.67±5.16	−1.3±3.27	<0.0001
Within group p-value	<0.001	0.055	
% of body weight	6.44%	1.48%	<0.0001
Loss >5% of body weight (%)	22 (59.5%)	6 (16.2%)	0.0001
Loss >10% of body weight (%)	12 (32.4%)	1 (2.70%)	0.0008
Loss >15% of body weight (%)	2 (5.4%)	0 (0.00%)	0.4932
**Changes in body mass index**	−2.08±1.91	−0.52±1.36	0.0001
Within group p-value	<0.001	0.008	
**Incidence of adverse events**			
Hypertension	0	0	-
Ischemic heart disease	0	0	-
Hemorrhagic stroke	0	0	-
Ischemic stroke	0	0	-

Continuous covariates are presented as the mean±standard deviation, while categorical covariates are presented as number (percentage). The p-values were calculated by student t-test and Pearson's chi-squared test for continuous and categorical covariates, respectively. Paired t-tests were used to compared the within-group weight and BMI changes.

Abbreviations: LA, laser acupuncture; PSM, propensity score matching
